# Vacuolar Processing Enzymes in Plant Programmed Cell Death and Autophagy

**DOI:** 10.3390/ijms24021198

**Published:** 2023-01-07

**Authors:** Karolina Wleklik, Sławomir Borek

**Affiliations:** Department of Plant Physiology, Faculty of Biology, Adam Mickiewicz University, Poznań, Uniwersytetu Poznańskiego 6, 61-614 Poznań, Poland

**Keywords:** Atg genes, Atg proteins, autophagic bodies, caspases, protease, tonoplast, vacuole

## Abstract

Vacuolar processing enzymes (VPEs) are plant cysteine proteases that are subjected to autoactivation in an acidic pH. It is presumed that VPEs, by activating other vacuolar hydrolases, are in control of tonoplast rupture during programmed cell death (PCD). Involvement of VPEs has been indicated in various types of plant PCD related to development, senescence, and environmental stress responses. Another pathway induced during such processes is autophagy, which leads to the degradation of cellular components and metabolite salvage, and it is presumed that VPEs may be involved in the degradation of autophagic bodies during plant autophagy. As both PCD and autophagy occur under similar conditions, research on the relationship between them is needed, and VPEs, as key vacuolar proteases, seem to be an important factor to consider. They may even constitute a potential point of crosstalk between cell death and autophagy in plant cells. This review describes new insights into the role of VPEs in plant PCD, with an emphasis on evidence and hypotheses on the interconnections between autophagy and cell death, and indicates several new research opportunities.

## 1. Introduction

Vacuolar processing enzymes (VPEs), which can also be named asparaginyl endopeptidases (AEPs), legumains, or colloquially, plant caspases, as they perform caspase-1-like/YVADase activity, are widespread in the plant kingdom. Their occurrence has been found in lower and higher plants [1]. VPEs are common in animals as well, but differ significantly, as only one isoform is active [2,3] and localized in the endolysosomal system [4]. Initially, plant VPEs were found in seeds, but their expression also occurs in vegetative organs [5]. VPEs are cysteine proteases and members of the C13 family (EC 3.4.22.34) with the ability to cleave peptide bonds on the C-terminal side of both asparagine and aspartic acid residues by their catalytic cysteine residue [1,5,6]. Such a proteolytic modification is required for many vacuolar pro-proteins for their maturation or activation [7]. VPEs are synthesized as a precursor form folded in the endoplasmic reticulum (ER) [6]. Then, zymogens are subjected to autoactivation by the successive removal of C- and N-terminal pro-peptides [8] in an acidic environment, such as in a vacuole with pH around 4.0 to 5.5 [2]. Thus, VPEs, which are autocatalytic themselves, may be considered as up-regulators of other vacuolar hydrolases [9]. It is assumed that by activating these vacuolar hydrolases, VPEs execute programmed cell death (PCD) [1]. Moreover, as key proteases in the vacuole, they potentially participate in the late stages of autophagy, e.g., degradation of the autophagic bodies [10,11,12]. This review provides new insights into the role of VPEs in plant PCD and autophagy with an emphasis on potential crosstalk between these two processes.

## 2. Classification of VPEs

Analysis of the *Arabidopsis thaliana* genome has revealed four VPEs (αVPE, βVPE, γVPE, and δVPE) that were previously divided into three subfamilies based on the homology and expression pattern: seed-type (βVPE), vegetative-type (αVPE, γVPE), and uncharacterized-type (δVPE) [13]. However, this initial classification of VPEs was not without discrepancies; for example, it was reported that although βVPE plays the main role in the processing of storage proteins in *Arabidopsis thaliana*, vegetative VPEs can also be expressed in the embryo during seed maturation [14]. The new classification is based on an analysis of the phylogenetic tree of VPE proteins, as different clades are characterized by the occurrence of different isoforms. The simplified classification is as follows: in angiosperms, there are two major types, γVPE and βVPE, whereas in gymnosperms the distinction between these two types of VPEs does not occur. These two types of VPEs are also found in monocots and basal eudicots. Subsequent clades belonging to core eudicots are characterized by the occurrence of δVPE. The occurrence of αVPE has only been confirmed in plants belonging to the Brassicaceae family. This phylogenetic classification is not perfect, however, as some data are missing [15]. Nevertheless, it can be recognized that four *Arabidopsis thaliana* genes of VPEs have been generated by three gene duplication events, which started with the evolution of angiosperms from gymnosperms. The first gene replication event has been well studied in the most recent common ancestor of the living flowering plant *Amborella trichopoda* [16]. Based on the genome-wide identification of VPE genes, it can be assumed that their number is not constant among species. The genome of the model plant *Arabidopsis thaliana* contains four VPE genes [5,17], the pear (*Pyrus*) genome contains eight VPE genes (named from *PbrVPE1* to *PbrVPE8*) [18], and the apple (*Malus*) genome contains twenty VPE genes (*MdVPE*) [19].

## 3. Functions of VPEs

VPEs are regulators of various critical processes in the plant life cycle. Primarily, it has been found that VPEs are responsible for the maturation of seed storage proteins such as 2S albumin and 12S globulin [20]. Now it is known that they participate in other developmental processes [21,22,23,24,25,26], senescence [27,28,29], and environmental stress responses [28,30,31,32]. During development, senescence, and plant responses to environmental stimuli, both autophagy [11] and PCD can be initiated [33]. Upregulation of VPEs occurs in various types of PCD. VPE involvement has been widely demonstrated during developmental PCD (dPCD), including seed coat formation in angiosperms [21], xylogenesis [23], development of the root velamen radicum in the epiphytic orchid *Cymbidium tracyanum* [25], development of pollen and tapetal cell degradation in *Arabidopsis thaliana* [24,26], leaf morphogenesis of the lace plant (*Aponogeton madagascariensis*) [34], and degradation of aleurone layers in rice (*Oryza sativa*) [22,30]. One of the best-known types of PCD regulated by VPEs is the hypersensitive response (HR), which was first observed in VPE-silenced *Nicotiana benthamiana* infected with tobacco mosaic virus (TMV) [35]. Furthermore, they take part in PCD induced by various abiotic stresses [19,28,30,36,37,38,39] and are even called executors of plant PCD. On the other hand, their role in the late stages of autophagy, i.e., degradation of autophagic bodies, is only presumed. Nevertheless, new data on the involvement of autophagy in PCD have appeared recently, and VPEs are considered an important part of this relationship [25,32].

Besides protease activity, the ligase activity of VPEs has also been observed [15,40]. The protein ligation activity of VPEs has been studied on the two-chain hybrid form of γVPE (*AtLEGγ*). It contains the C-terminal pro-domain LSAM (legumain stabilization and activity modulation), which modulates its activity and provides stability at neutral pH. Under such conditions, ligase activity rather than protease activity is favored [2]. Meanwhile, some VPEs preferably exhibit ligase or protease activity regardless of pH. VPEs isolated from *Clitoria ternatea*, named butelase 1 and butelase 2, perform predominantly ligase activity at a mildly acidic pH and protease activity at neutral pH, respectively [40]. The ligase activity allows VPEs to form cyclic peptides [15,41,42,43]. It has been observed that some VPEs may perform greater protein cyclization activity than others. Among four VPEs (*PxAEP1*, *2*, *3a*, and *3b*) found in petunia (*Petunia*), PxAEP3b has been characterized with the most efficient ability to produce cyclic peptide kalata B1 (kB1). PxAFP3a was found to be significantly less effective in kB1 cyclic formation despite having a sequence very similar to that of PxAFP3b [44].

## 4. VPEs Are Executors of Plant PCD

Plant PCD is classified into two types: autolytic and non-autolytic. Autolytic PCD contributes to tonoplast rupture and destruction of the cytoplasm, while non-autolytic PCD takes place when rupture of the tonoplast is observed but rapid cytoplasm clearance does not occur [45]. Autolytic PCD is also associated with chromatin condensation and an increase in vacuolar volume. This type of PCD occurs during developmental processes and plant responses to abiotic stress, whereas non-autolytic PCD is also associated with the swelling of organelles and is mainly observed under biotic stress [46]. Taking into consideration the internal and external stimuli, two types of PCD can be distinguished: developmental (dPCD) and environmental (ePCD). PCD types are regulated by different classes of transcription factors (TFs), which can promote or suppress cell death. Members of NAC, the largest TF family, are important during both ePCD [47,48] and dPCD [33]. However, both types of PCD are characterized by processes that take place in a similar way, including calcium signaling, generation of reactive oxygen species (ROS), and induction of VPE activity [33].

Caspases, regulators of PCD in animals, have not been found in plants. Nevertheless, plant cells contain proteases that exhibit caspase-like activity, including VPEs [1,49]. Besides VPEs, other PCD-promoting cysteine proteases are present in plants. These enzymes include metacaspases, which are divided into three types [50]. Types I and II occur widely in the plant kingdom, whereas type III (GtMC2) has so far only been found in the genome of the algae *Guillardia theta* [51]. Although metacaspases are regulators of some types of PCD [52], unlike caspases they are substrate-specific for arginine and lysine residues, and therefore, their activity should not be called caspase-like [45]. This specific caspase-like activity can be mediated in plants by serine proteases such as phytaspases and saspases [49]. Phytaspases and saspases are subtilisin-like proteases with the ability to hydrolyze several peptide-based caspase substrates. Despite being located in the extracellular space, it has been observed that saspases’ activity upon PCD induction is significantly higher, and phytaspases even translocate to the cytoplasm [53]. It is presumed that saspases regulate the proteolysis of RuBisCO [54], whereas phytaspases are known for regulating HR induced by TMV and regulating PCD during oxidative and osmotic stresses [55]. Other proteases contributing to PCD are papain-like cysteine proteases (PLCPs) such as cathepsins [56]. Cathepsin B, which performs caspase-3-like/DEVDase activity, can be blocked by caspase-3 inhibitors, and therefore, its inhibition downregulates PCD [57]. The *Arabidopsis thaliana* cathepsin B mutant showed no difference in tonoplast rupture mediated by VPEs. However, this manipulation contributed to decreased ER stress-induced PCD and decreased accumulation of ROS. Likewise, the *Arabidopsis vpe-null* mutant also showed a decreased cell death rate, but cathepsin B activation remained unchanged, meaning that VPEs are not required for its maturation. These observations reveal that cathepsin B and VPEs act in parallel to execute PCD, but independently [58].

VPEs are involved in the execution of a variety of plant PCDs possibly by up-regulation of various vacuolar hydrolytic enzymes. This ability makes them counterparts of animal caspases [1]. There are few evolutionary links between VPEs and caspases, as they show only about 15% sequence homology [4]. Regardless, the crystal structure of these two enzymes is similar. Both are characterized by a topologically equivalent central six-stranded β-sheet (β1β6), flanked by five major α-helices (α1–α5). Differences in the structure are also observed: for example, an approximately 30-aa insertion between strand β2 and helix α2. Moreover, plant VPEs are active as monomeric forms, whereas caspases are active as dimeric forms [59]. VPEs are specifically compared to animal caspase-1, as they both perform YVADase activity [1,34]. VPEs such as caspase-1 cleave peptide bonds on the C-terminal side of asparagine residues but with an extra ability to cleave peptide bonds of aspartic acid residues. Interestingly, VPEs cannot recognize aspartic acid residues other than those included in the YVAD sequence. Thus, aspartic acid residues included in the DEVD sequence, which is a caspase-3 substrate, are not cleaved by VPEs [1]. The key difference between these enzymes seems to be localization, as caspase-1 is located in the cytoplasm. Therefore, cell deaths mediated by these two enzymes differ significantly [60]. The molecular mechanism of VPE-mediated tonoplast rupture is unknown. It is presumed that VPEs process other hydrolases and initiate the proteolytic cascade followed by PCD [1].

## 5. Role of VPEs in PCD under Biotic Stress

Local PCD, also called the hypersensitive response (HR), is a radical but effective plant method to combat various biotic stressors such as viruses, bacteria, and fungi. Rapid death of cells at the site of pathogen infection prevents it from spreading in the host plant [61]. HR mediated by VPEs was first observed on VPE-silenced *Nicotiana benthamiana* infected with TMV. In VPE-non-silenced plants, pathogen attack was related to increased expression and translation of VPEs in the infected leaves. Moreover, ultrastructural images showed disintegration of the tonoplast in the cells of VPE-non-silenced plants, whereas in cells of VPE-silenced plants, vacuole morphology remained unchanged. These ultrastructural analyses have shown the contribution of VPEs to vacuole collapse during PCD [35]. Further research has shown that VPEs are involved in plant defense against such pathogens as the bacterium *Erwinia amylovora* [62], nematode *Heterodera filipjevi* [63], the oomycetes *Hyaloperonospora arabidopsidis* [64] and *Phytophthora parasitica* [65], and the fungi *Phaeoisariopsis personata* [66], *Fusarium oxysporum* (FocTR4) [67] and *Botryosphaeria dothidea* [68].

## 6. Role of VPEs in PCD Induced by Abiotic Stress

VPEs have been found to mediate ePCD induced by several abiotic stresses. Genome-wide analysis of the apple (*Malus*) genome has shown the presence of twenty genes coding for VPEs (*MdVPEs*), which have been distinguished into four groups based on *Arabidopsis thaliana* types: *MdαVPEs*, *MdβVPEs*, *MdγVPEs*, and *MdδVPEs*. Expression patterns of eighteen *MdVPEs* were examined under abiotic stresses such as salinity, cadmium treatment, low temperature, and drought. Each of the above-mentioned stresses increased the expression of some *MdVPEs*; however, during salinity, eighteen examined *MdVPEs* were up-regulated. It has also been shown that different groups respond specifically to different stresses, as three of five *MdγVPEs* were more sensitive to drought and salinity than cadmium and low temperature [19]. On the other hand, genome-wide analysis of upland cotton (*Gossypium hirsutum*) showed the presence of thirteen genes coding for VPEs (*GhVPEs*). Three of these showed increased expression under waterlogging and salinity. In detail, VPEs of upland cotton whose expression increased during these abiotic stresses were γ- and δVPE-like [39]. Salinity-induced PCD has also been studied in rice (*Oryza sativa*), in which four genes of VPEs (*OsVPEs*) were found. In particular, *OsVPE3* mediated salinity-induced PCD, as its silencing increases plant tolerance to this kind of stress. Moreover, it has been demonstrated that PCD prevention by silencing *OsVPE3* is related to the suppression of tonoplast rupture [37]. Salinity also increased the expression of γVPE in alfalfa (*Medicago sativa*) root meristem. Interestingly, melatonin treatment reduced ROS formation and decreased γVPE gene expression, which prevented salinity-induced ePCD. The pro-survival mechanism of melatonin is thought to be related to upregulation of uncoupling proteins 1 and 2 (UCP1 and UCP2) and Bax inhibitor-1 (BI-1) genes. UCPs probably mediate a decrease in electron leakage and ROS formation in plant mitochondria, whereas BI-1 (inhibitor of pro-apoptotic Bax protein) regulates Ca^2+^ homeostasis [38]. Both ROS and Ca^2+^ are signal messengers that can participate in the MPK activation cascade. MPK6 was found to positively regulate γVPE expression in *Arabidopsis thaliana* seedlings during abiotic stress [36]. Therefore, melatonin may contribute to the initiation of a molecular cascade that leads to a decreased expression of γVPE. ePCD can also be induced by low or high temperatures. In *Arabidopsis thaliana*, increased gene expression and enzyme activity of γVPE were observed after heat shock induction. Silencing γVPE, as in the case of rice, also contributed to suppression of tonoplast rupture. Moreover, a relation between γVPE and mitogen-activated protein kinase (MPK6) was demonstrated. The application of MPK6 inhibitor during heat shock contributed to a decrease in gene expression and enzyme activity of γVPE. Similar relations have been observed with mutants lacking MPK6. Additionally, *Arabidopsis* mutants overexpressing MPK6 showed an increase in γVPE gene expression and enzyme activity resulting in a significant decrease in seedling fresh weight in comparison to the wild type. Therefore, MPK6 may be considered as a positive regulator of γVPE [36]. The members of the NAC family, transcription factors of PCD, also regulate the expression of VPEs. *GmNAC30/GmNAC81* from soybean (*Glycine max*) affected the expression of VPEs by directly activating their promoters under ER- and osmotic stress-induced PCD [69]. Molecular manipulation of *GmNAC81* altered the plant response to stress. Overexpression of *GmNAC81*, through the mediation of VPEs, increased the sensitivity of plants to drought [28]. VPEs have also been found to execute sugar starvation-induced ePCD in tobacco BY-2 cells. Moreover, in this case for the first time, it was observed that VPEs are translocated from the ER to the vacuole through autophagosomes [32].

## 7. Autophagy Contribution to Cell Death

**Figure 1:**
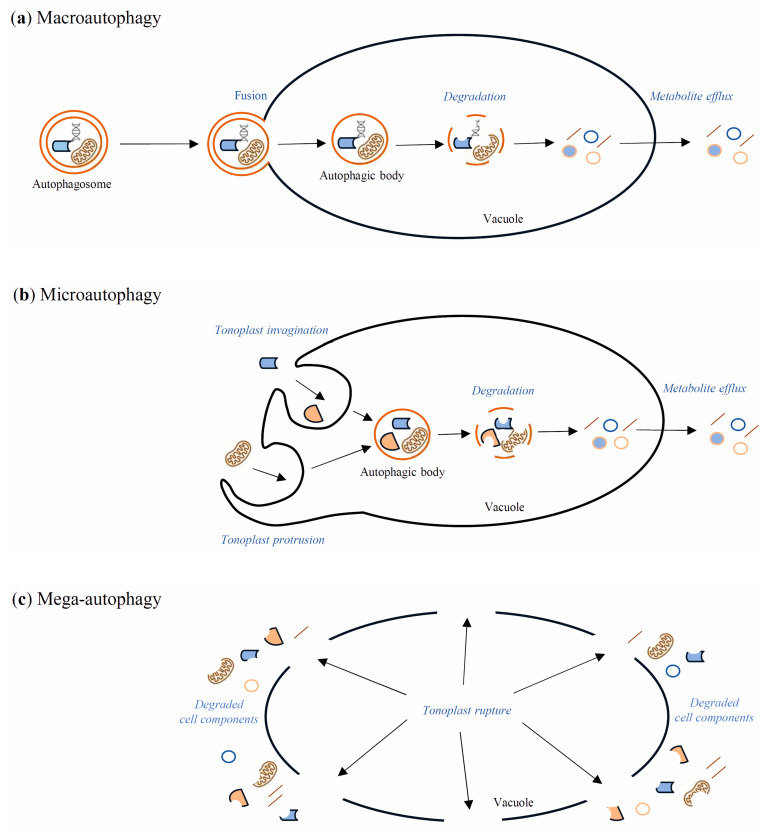
Schematic diagram of macroautophagy (**a**), microautophagy (**b**), and mega-autophagy (**c**) in plants. During macroautophagy, cargo intended for degradation is transported to the vacuole inside an autophagosome. The outer membrane of the autophagosome fuses with the tonoplast, while the internal autophagosome membrane and the cargo create an autophagic body inside the vacuole. The autophagic body is rapidly degraded by vacuolar hydrolases, which allow for the recycling of metabolites. During microautophagy, the autophagosome is not formed, but cell components intended for degradation enter the vacuole through the tonoplast invagination or tonoplast protrusion. Inside the vacuole, there arise the autophagic bodies, which, as in macroautophagy, are degraded by vacuolar hydrolases. Mega-autophagy differs significantly from macro- and microautophagy, as cell elements are not transported to the vacuole for degradation. Instead, the vacuole membrane is destroyed, and subsequently cell death occurs.

Autophagy is the evolutionarily well-conserved process of cell self-eating occurring in yeasts, animals, and plants. Through the autophagy pathway, cellular components such as protein complexes and organelles are degraded. Moreover, bacteria and viruses can also be degraded in the infected cells through this process [70]. Autophagy takes place in all life stages of the plant, including development, senescence, and cell death [12,71]. Under normal development and growth conditions, the insensitivity of autophagy is relatively low—basal. Then, it works as a quality control mechanism to degrade and recycle unwanted or damaged cellular components [72]. However, it remarkably increases during biotic and abiotic stresses such as nutrient deficiency, drought, salinity, heat, oxidation, and pathogen attack [12]. In yeast, autophagy is regulated by over forty AuTophaGy-related (Atg) genes, which have also been found in animals and plants [73]. These genes encode Atg proteins, which play many roles during autophagy processes. For example, the Atg1/Atg13 kinase complex is essential for autophagy initiation by the target of rapamycin (TOR) signaling pathway [74]. TOR, a serine/threonine kinase, negatively regulates autophagy in response to many environmental stimuli [75], whereas the sucrose nonfermenting-1-related protein kinase 1 (SnRK1) is the central kinase complex, which positively regulates autophagy by activation of Atg1 kinase [76]. Autophagy occurs in both selective and non-selective ways. The selective form of autophagy takes place when only particular cell components are degraded, for example, mitochondria (mitophagy) or peroxisomes (pexophagy) [77,78,79]. Due to the differences in the delivery of the cargo intended for autophagic degradation, the following types of autophagy in plants are distinguished: macroautophagy, microautophagy, and mega-autophagy [11,80,81,82]. Macroautophagy (Figure 1a) is the best-known type of autophagy. It starts with the formation in the cytoplasm of a cup-shaped structure named a phagophore. The phagophore elongates until it is surrounded by cell components intended for degradation. A vesicle with a bilayer double-membrane, containing cargo intended for degradation, is called an autophagosome. These stages of macroautophagy are similar in yeasts, plants, and animals. The next stage, which is directing the autophagosome to the lytic cell compartments, is similar in yeasts and plants but distinguished from animals. Namely, in yeasts and plants, the autophagosome is directed to the vacuole, where it fuses with the tonoplast by its outer membrane. The unaffected internal membrane of the autophagosome with the cargo inside creates an autophagic body inside the vacuole. In animals, the autophagosome is directed to the lysosome, where they fuse, creating an autolysosome. Finally, in both the vacuole and the autolysosome, cargo is degraded by lytic enzymes [11,79,83]. Microautophagy (Figure 1b) is an autophagy pathway in which autophagosomes are not formed. Cell elements intended for degradation enter the lysosome or vacuole by membrane invagination or protrusion of these organelles [11,79]. Mega-autophagy (Figure 1c) has only been observed in plants, and it is perceived as massive cytoplasm destruction that occurs during dPCD and abiotic stress-induced ePCD. Nonetheless, none of the Atg genes are involved in mega-autophagy, and cellular components are not directed to the vacuole for degradation [84,85].

**Figure 2:**
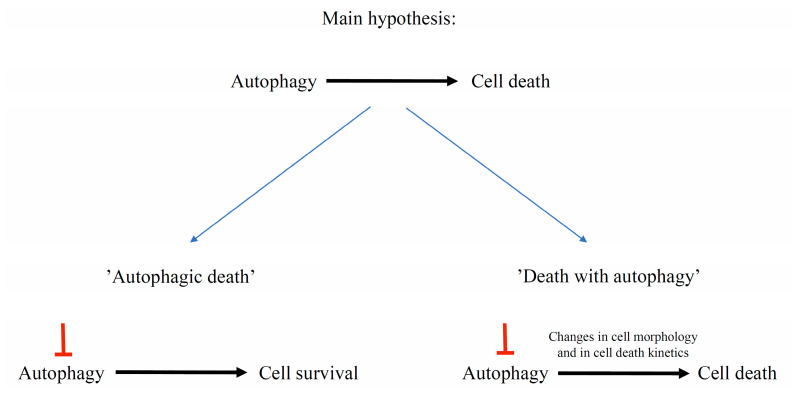
To determine whether autophagy acts in a cell pro-death or pro-survival manner, an experimental approach based on inhibition of autophagy is needed. “Autophagic death” occurs when inhibition of autophagy contributes to cell survival, whereas “death with autophagy” occurs when inhibition of autophagy, for example, delays cell death, but finally it will occur.

Autophagic cell death (ACD) is the second form of animal PCD. It is associated with increased numbers of autophagosomes, autolysosomes, and small lytic vacuoles [46]. Autophagic death is a controversial idea that has been discussed and debated many times. Autophagy is considered as a two-faced process: it can ensure cell survival as well as promote cell death [86]. However, it is difficult to distinguish when the occurrence of autophagic-related structures and recruitment of Atg genes function with the aim of cell survival and, conversely, when the aim is cell death. To solve this problem, it has been proposed to define “autophagic death” as when inhibition of autophagy contributes to long-term cell survival. In contrast, “cell death with autophagy” should be defined when inhibition of autophagy does not determine the subsequent death of the cell, but may change its morphology and delay the process (Figure 2) [87]. Therefore, crosstalk between these two processes remains important to study. Many genes are involved in both autophagy and cell death in animal models [86]. Although our knowledge of this subject in plants is limited, a few examples of cell death with autophagy have been described, and VPEs, as proteases associated with cell death execution in plants, may be an important factor connected with the pro-death or pro-survival role of plant autophagy [25,32].

The initial degradation of cellular components by autophagy may be important for subsequent dPCD in plants. During PCD-dependent development of the root velamen radicum in the epiphytic orchid *Cymbidium tracyanum*, five genes of VPE, eight genes related to autophagy, and two genes of metacaspases were upregulated [25]. The differentiation of tracheary elements of the xylem is also a process in which dPCD and autophagy come together. It has been found that autophagy-related small GTP binding protein RabG3b and atg5 may be involved in xylem development of *Arabidopsis thaliana* [88]. The potential involvement of Atg genes in dPCD during xylogenesis has also been evaluated in the root of *Populus trichocarpa*. Increased expression levels of Atg8h, Atg11, and Atg18d genes were found in the isolated secondary xylem cells in comparison to the primary stem cells, implying that activation of these genes may be significant to dPCD [89]. Similarly, autophagy and PCD coexist in senescing barley (*Hordeum vulgare*) leaves. Among two VPEs (*αVPE* and *VPE2c*) and four Atg genes (*Atg4*, *Atg6*, *Atg8*, *Atg9*), the expression of αVPE and all Atg genes increased after ten days of senescence [90]. The involvement of autophagy in dPCD was also found in the root cap of *Arabidopsis thaliana*. Mutation of key autophagy genes Atg2, Atg5, and Atg7 contributed to the delay of dPCD and subsequent protoplast clearance in some cells of the root cap [91].

The potential involvement of autophagy in cell death is not only characteristic for dPCD. Atg6/BECLIN-like protein is required to limit HR to infected tissues in *Arabidopsis thaliana* attacked by *Pseudomonas syringae* pv. *tomato* (Pst). In yeast, Atg6/Vps30 is one of the key autophagy proteins, as it is involved in autophagosome formation [92]. On the other hand, *atg7-1* and *atg9-1* knockout mutants of *Arabidopsis thaliana* showed the pro-death function of autophagy during HR, as such manipulation contributed to cell death inhibition [93]. In addition, it has been shown that pathogen effectors, for example, HopF3, affect Atg proteins and through that action modulate autophagy to enhance virulence [94].

**Figure 3:**
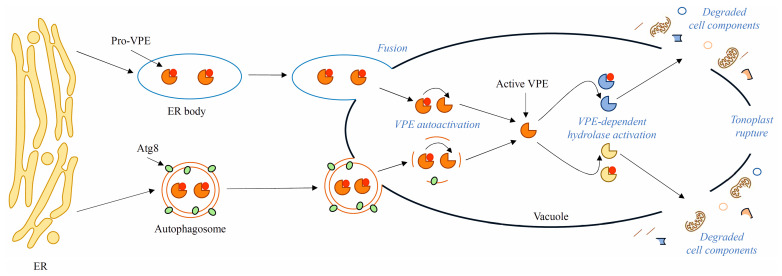
Possible ways of transport of VPEs in plant cells. Premature VPEs are synthesized in the endoplasmic reticulum (ER) and then are translocated to the vacuole through spindle-shaped ER bodies with single membranes or through autophagy inside autophagosomes tagged with Atg8. After fusion with the tonoplast, pro-VPEs are subjected to autoactivation in acidic pH inside the vacuole. It is presumed that mature VPEs process other vacuolar hydrolases, which contribute to tonoplast rupture and cell death.

VPEs may be the point of crosstalk between autophagy and PCD. Simultaneous carbon starvation and treatment with the autophagy inhibitor concanamycin A of tobacco BY-2 cells expressing *StVPE1*-GFP resulted in accumulation in the vacuole of both autophagic bodies and labeled *StVPE1*. Moreover, colocalization of VPE and Atg8IL anchored in the outer membrane of autophagosome has been demonstrated. Silencing of Atg4, which is essential for Atg8 processing, contributed to decreased VPE activity and cell death rate. Taken together, the evidence implies that VPE translocates through the autophagy pathway to the vacuole, where it executes cell death (Figure 3) [32]. On the other hand, it has previously been shown that γVPE can be translocated through ER bodies (Figure 3) to the vacuole to promote stress-induced cell death in young seedlings of *Arabidopsis thaliana* [95,96]. Nevertheless, it has also been found that dPCD of pericarp cells in wheat (*Triticum*) grains coexists with the autophagy pathway, as silencing Atg8 inhibited dPCD and caused the formation of small premature grains with a thick pericarp layer [97]. However, by the manipulation of autophagy with inhibitors and accelerants such as concanamycin A, wortmannin, and rapamycin, autophagy was found to promote cell survival rather than cell death in the lace plant (*Aponogeton madagascariensis*). Direct involvement of autophagy in dPCD has not been implicated, but on the other hand, the number of Atg8-positive points in the cells increased as cell death progressed [98]. In conclusion, it seems that ePCD and dPCD pathways may be strongly associated with autophagy processes, and VPE activity may be dependent on autophagy regulators such as Atg8. The results described here do not explain fully the dependencies between autophagy and cell death, but rather constitute an introduction to future research.

## 8. Presumed Role of VPEs in Late Stages of Autophagy

Plant autophagy is often studied by homology to yeast autophagy, as autophagy is a highly conserved process; for example, the key molecular components of autophagy such as Atg genes and proteins or the TOR kinase complex were first observed and described in yeast, and then found in animals and plants. Therefore, homology studies may help to fill the gaps in knowledge about the molecular mechanisms of plant autophagy [11]. In yeast, the degradation of autophagic bodies is processed by the following enzymes: proteinase B (Prb1), proteinase A (Pep4) [11,99], and Atg15 possessing lipolytic activity [11,100,101]. Additionally, Atg42/Ybr139w and its homolog carboxypeptidase Y (Prc1) are likely also involved in the degradation of autophagic bodies in yeast [11,102]. In *Saccharomyces cerevisiae* mutants lacking Prc1, no significant accumulation of autophagic bodies in the vacuoles was observed, indicating that Prc1 is not critical for autophagic bodies’ degradation [11,99]. Although the knockout of both Prc1 and Atg42/Yrb139w disturbed the breakdown process, the mechanism of their function is not understood [11,102]. Another protein potentially involved in degradation of the autophagic bodies in yeast is the putative vacuolar permease Atg22. It does not directly regulate this process like proteinase A, whereas the kinetic delay in autophagic body breakdown has been observed in Atg22 mutants [11,103].

VPEs, with their ability to activate other proteases, are candidate counterparts of yeast proteinases, especially proteinase A. Proteinase A is an up-regulator of other proteases, including proteinase B [99] and carboxypeptidase Y [104,105]. It has been demonstrated that VPE from castor bean (*Ricinus communis*) can replace proteinase A and effectively process the proenzyme of carboxypeptidase Y (CPY) to the mature form in yeast cells [105]. Moreover, γVPE of *Arabidopsis thaliana* is responsible for the maturation of CPY, which is a homolog of yeast Prc1 [9]. Taken together, the assumptions that VPEs participate in the degradation of autophagic bodies are not baseless and have been presented several times in the literature [10,11,12]. However, no evidence confirming such a role of VPEs in autophagy has been published so far.

## 9. Conclusions and Future Perspectives

The participation of VPEs in various types of PCD has been well proven. These cysteine proteases, among others, are responsible for tonoplast rupture, which is followed by the outflow of vacuolar hydrolases to the cytoplasm and cell death, although it is not yet known which particular proteases (both VPEs or other proteases activated by VPEs) are involved in this process. The number of VPE genes depends on the species, and it is also poorly understood which (and why) different VPEs execute PCD during ontogenesis and under changing environmental conditions. Therefore, more research on the role of particular VPEs in the activation of vacuolar hydrolases and the involvement of these enzymes in autophagy and PCD is needed.

Autophagy was first observed in the 1950s [106]. Decades of research have revealed the key importance of autophagy in plant development and responses to internal and external stimuli. Nonetheless, many aspects, for example, the late stages of autophagy, i.e., degradation of autophagic bodies and metabolite efflux from the vacuole to the cytoplasm, have been overlooked in the research, and the knowledge about these stages of autophagy in plants is vestigial. The process of degradation of autophagic bodies in yeast occurs with the participation of several known enzymes, whereas in plants, only VPEs are taken into consideration as potentially involved in this process. However, it is not clear how VPEs would distinguish their pro-death activity during PCD from pro-survival activity during autophagy and limit their role only to the initiation of autophagic body degradation. Moreover, the links between autophagy and PCD are still poorly understood. The evidence that VPEs may be delivered to the vacuole by the autophagy pathway seems to be a good reference point for future investigations. The Atg8 gene family, which encodes ubiquitin-like proteins required for autophagosome formation, should also be examined as a potential point of crosstalk between autophagy and cell death.

In conclusion, the data presented in this review did not fill the gaps in knowledge about the role of VPEs in plant PCD and autophagy but did uncover several new research opportunities.

## Data Availability

Not applicable.

## Abbreviations

Atg	Autophagy-related genes or proteins
BI-1	Bax inhibitor-1
dPCD	Developmental programmed cell death
ePCD	Environmental programmed cell death
HR	Hypersensitive response
kB1	Kalata B1
LSAM	Legumain stabilization and activity modulation pro-domain
MPK6	Mitogen-activated protein kinase 6
NAC	NAM, ATAF1/2, and CUC transcription factors
Pep4	Proteinase A
Prb1	Proteinase B
Prc1	Carboxypeptidase Y
ROS	Reactive oxygen species
RuBisCO	Ribulose-1,5-bisphosphate carboxylase/oxygenase
SnRK1	Sucrose nonfermenting-1-related protein kinase 1
TFs	Transcription factors
TOR	Target of rapamycin serine/threonine-protein kinase
UCP	Uncoupling protein
VPEs	Vacuolar processing enzymes
Vps30	Vacuolar protein sorting-associated protein 30
Ybr139	Putative serine carboxypeptidase YBR139W
